# Supplementary material for: Classification and Regression Trees analysis identifies patients at high risk for kidney function decline following hospitalization
Source: PLoS One. 2025 Jan 31;20(1):e0317558. doi: 10.1371/journal.pone.0317558 (PMC11785296; doi:10.1371/journal.pone.0317558)
Supplement: S3 Fig — (DOCX) [file pone.0317558.s003.docx]

**S3 Fig.** **Random Forest for fast eGFR decline in the COVID positive subset (N = 260)**


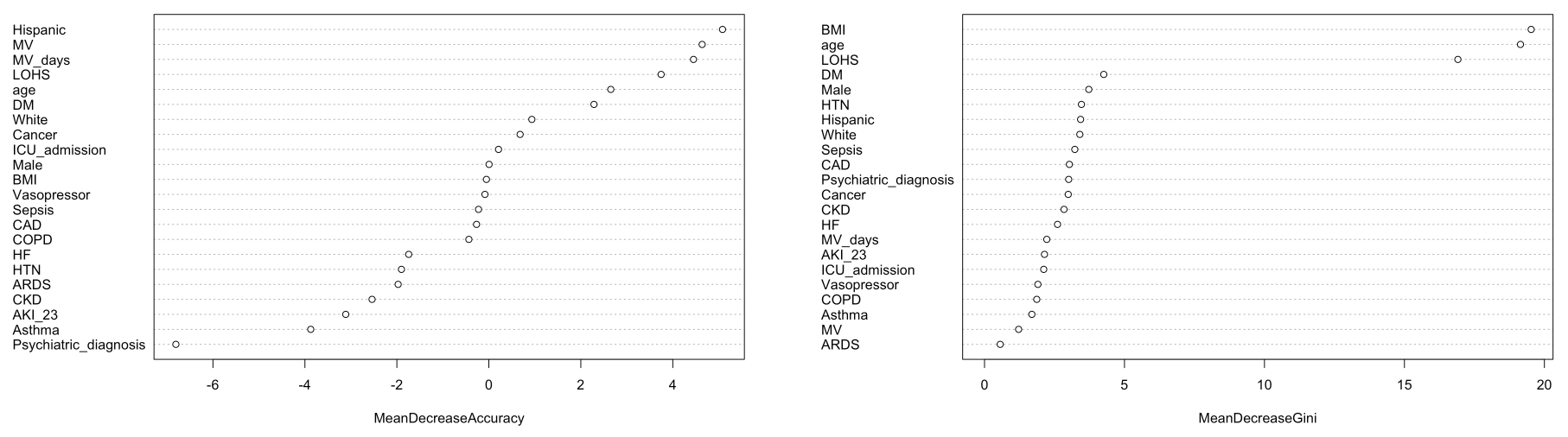


**Legend:**

Abbreviations: DM = diabetes mellitus, HF = heart failure, CKD = chronic kidney disease, COPD = chronic obstructive pulmonary disease, HTN = hypertension, CAD = coronary artery disease, BMI = Body Mass Index, LOHS = length of hospital stay, ICU admission = intensive care unit admission, MV = mechanical ventilation, ARDS = acute respiratory distress syndrome, AKI = acute kidney injury, COVID-19 = Corona virus disease 2019.

Mean decrease accuracy quantifies a variable's predictive power by measuring model accuracy loss when the variable is removed, while mean decrease Gini assesses a variable's ability to create pure, well-separated nodes during tree splitting.

The importance of variables was ranked based on mean decrease accuracy and mean decrease Gini of RF analysis. The top 10 variables from both metrices were used in the following CART tree and Multivariate logistic regression analysis.
