# Supplementary material for: Classification and Regression Trees analysis identifies patients at high risk for kidney function decline following hospitalization
Source: PLoS One. 2025 Jan 31;20(1):e0317558. doi: 10.1371/journal.pone.0317558 (PMC11785296; doi:10.1371/journal.pone.0317558)
Supplement: S12 Fig — (DOCX) [file pone.0317558.s012.docx]

**S12 Fig.** **CART decision tree for fast eGFR decline in the COVID negative subgroup of the PSM matched COVID-19 subset (N = 260)**


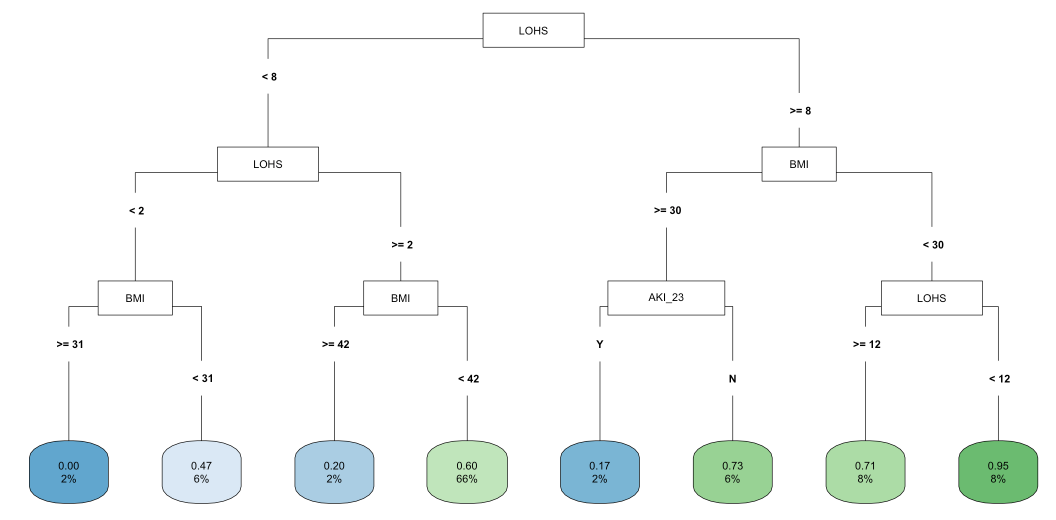


Legend: The number of observations in a terminal node was set as at least 2% of the sample size. The percentage mentioned in the terminal node is the % of patients of the starting cohort of the analyses. In each terminal node, the risk of fast eGFR decline (vs. not fast) ranges from 0.00 (lowest) to 1.00 (highest). The color of the terminal node represents the risk associated with the tree attached to each node, with the intensity of green color indicating a stronger risk, while intensity of blue color representing a lower risk. The maximum depth of the decision tree was set to be 3.
