# Supplementary material for: Classification and Regression Trees analysis identifies patients at high risk for kidney function decline following hospitalization
Source: PLoS One. 2025 Jan 31;20(1):e0317558. doi: 10.1371/journal.pone.0317558 (PMC11785296; doi:10.1371/journal.pone.0317558)
Supplement: S14 Fig — (DOCX) [file pone.0317558.s014.docx]

**SX b. Standardized Mean Differences (SMDs) Before and After Matching for COVID-19 Diagnosis Groups**


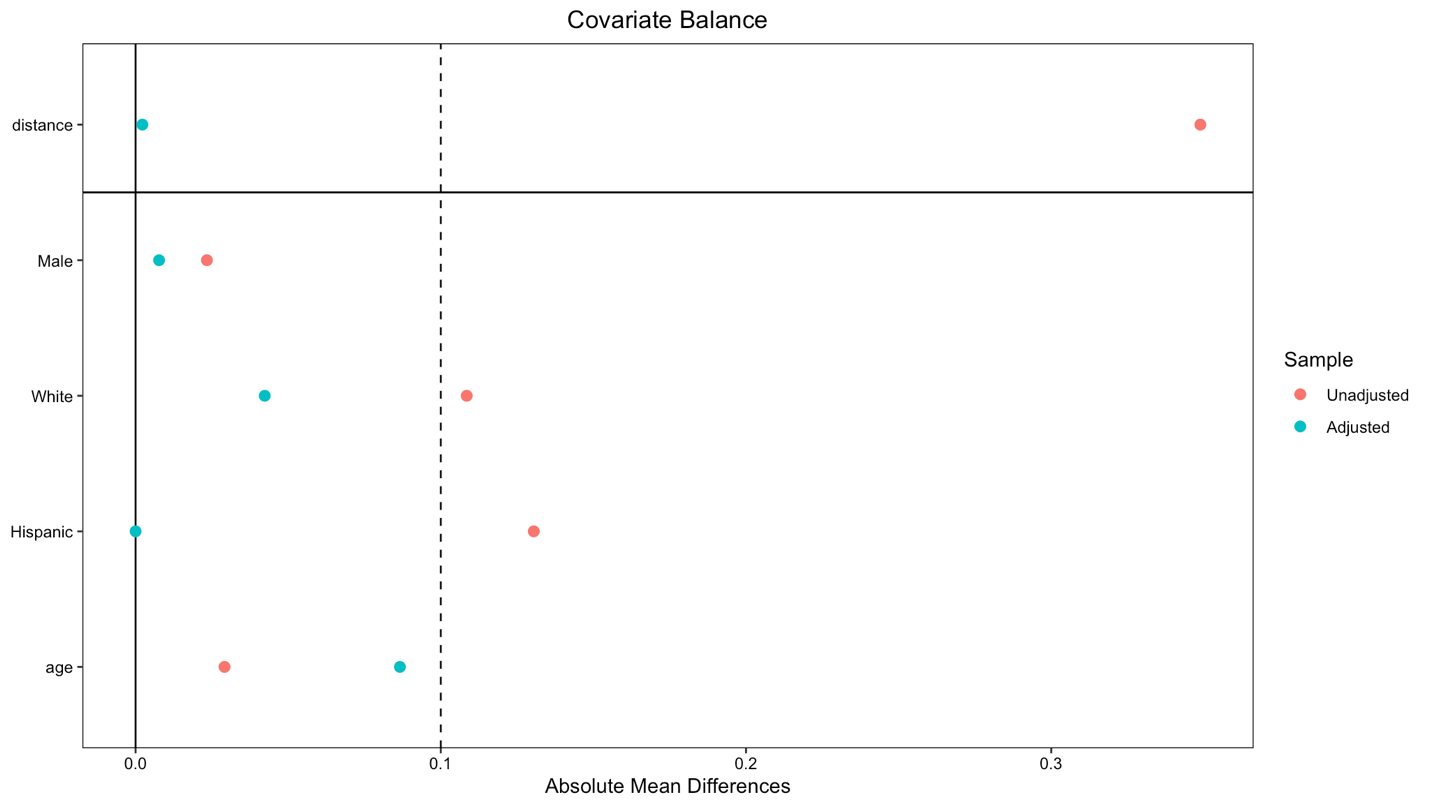


**Legend:**

absolute standardized mean differences (SMDs) for both the unadjusted (pre-matching) and adjusted (post-matching) datasets were calculated to assess balance. 0.10 was applied as the threshold for acceptable balance for adjusted SMDs.
