# Supplementary material for: Classification and Regression Trees analysis identifies patients at high risk for kidney function decline following hospitalization
Source: PLoS One. 2025 Jan 31;20(1):e0317558. doi: 10.1371/journal.pone.0317558 (PMC11785296; doi:10.1371/journal.pone.0317558)
Supplement: S1 Table — (DOCX) [file pone.0317558.s015.docx]

**S1 Table. Supplementary Table 1: Logistic regression for fast eGFR decline in the COVID negative subset (N = 1487)**

| **Variable** |  | **OR (univariable)** | **OR (multivariable)** |
| --- | --- | --- | --- |
| Vasopressor | 1 | 1.64 (1.27-2.12, ***) | **1.49 (1.14-1.95, **)** |
| LOHS | Mean (SD) | 1.03 (1.02-1.05, ***) | **1.02 (1.01-1.04, **)** |
| AKI_23 | 1 | 1.37 (0.95-2.00) | 0.97 (0.65-1.47) |
| White | 1 | 0.97 (0.77-1.24) | 1.01 (0.79-1.30) |
| Asthma | 1 | 0.92 (0.63-1.35) | 0.89 (0.60-1.33) |
| COPD | 1 | 0.89 (0.65-1.21) | 0.90 (0.65-1.24) |
| Male | 1 | 1.08 (0.88-1.33) | 1.04 (0.84-1.30) |
| HTN | 1 | 0.82 (0.66-1.01) | 0.84 (0.67-1.05) |
| CKD | 1 | 0.94 (0.73-1.20) | 0.95 (0.72-1.25) |
| ARDS | 1 | 0.32 (0.01-3.33) | 0.19 (0.01-2.13) |
| BMI | Mean (SD) | 1.00 (0.99-1.01) | 1.00 (0.99-1.01) |
| Age | Mean (SD) | 0.99 (0.99-1.00, *) | 0.99 (0.99-1.00) |
| Psychiatric diagnosis | 1 | 1.15 (0.93-1.42) | 1.13 (0.91-1.40) |
| DM | 1 | 0.96 (0.77-1.20) | 0.99 (0.78-1.26) |
| CAD | 1 | 0.98 (0.78-1.22) | 1.10 (0.85-1.41) |
| Cancer | 1 | 1.04 (0.81-1.34) | 1.00 (0.77-1.31) |

**Legend:**

Abbreviations: LOHS = length of hospital stay, COPD = chronic obstructive pulmonary disease, MV = mechanical ventilation, CKD = chronic kidney disease, HTN = hypertension, DM = diabetes mellitus, CAD = coronary artery disease, eGFR = estimated glomerular filtration rate.

The top variables form Random Forest analysis were selected for Logistic Regression analysis.

P-values < 0.05 were considered significant and were summarized with ‘*’, p-values < 0.01 were considered significant and were summarized with ‘**’, and p-values < 0.001 were considered significant and were summarized with ‘***’.
