# Supplementary material for: Classification and Regression Trees analysis identifies patients at high risk for kidney function decline following hospitalization
Source: PLoS One. 2025 Jan 31;20(1):e0317558. doi: 10.1371/journal.pone.0317558 (PMC11785296; doi:10.1371/journal.pone.0317558)
Supplement: S2 Table — (DOCX) [file pone.0317558.s016.docx]

**S2 Table. Logistic regression for fast eGFR decline in the COVID positive subset (N = 260)**

| **Variable** |  | **OR (univariable)** | **OR (multivariable)** |
| --- | --- | --- | --- |
| Hispanic | 1 | 0.76 (0.42-1.40) | 0.59 (0.29-1.21) |
| MV | 1 | 4.40 (1.66-15.23, **) | 1.44 (0.18-9.56) |
| MV days | Mean (SD) | 1.14 (1.04-1.34, *) | 1.02 (0.93-1.29) |
| LOHS | Mean (SD) | 1.04 (1.02-1.07, **) | 1.03 (1.00-1.07) |
| Age | Mean (SD) | 0.99 (0.98-1.01) | 0.98 (0.97-1.00) |
| DM | 1 | 1.86 (1.06-3.35, *) | **2.25 (1.21-4.30, *)** |
| White | 1 | 0.96 (0.56-1.62) | 1.06 (0.56-1.98) |
| Cancer | 1 | 0.58 (0.27-1.23) | 0.46 (0.20-1.06) |
| ICU admission | 1 | 2.65 (1.30-5.86, **) | 1.23 (0.43-3.90) |
| Male | 1 | 1.06 (0.63-1.76) | 0.82 (0.46-1.44) |
| BMI | Mean (SD) | 1.00 (0.97-1.03) | 0.98 (0.95-1.02) |
| HTN | 1 | 1.53 (0.92-2.56) | 1.49 (0.84-2.67) |
| Sepsis | 1 | 2.19 (1.19-4.21, *) | 1.66 (0.85-3.34) |
| CAD | 1 | 0.99 (0.55-1.83) | 1.09 (0.56-2.16) |

**Legend:**

Abbreviations: LOHS = length of hospital stay, COPD = chronic obstructive pulmonary disease, MV = mechanical ventilation, CKD = chronic kidney disease, HTN = hypertension, DM = diabetes mellitus, CAD = coronary artery disease, eGFR = estimated glomerular filtration rate.

The top variables form Random Forest analysis were selected for Logistic Regression analysis.

P-values < 0.05 were considered significant and were summarized with ‘*’, p-values < 0.01 were considered significant and were summarized with ‘**’, and p-values < 0.001 were considered significant and were summarized with ‘***’.
