# Supplementary material for: Classification and Regression Trees analysis identifies patients at high risk for kidney function decline following hospitalization
Source: PLoS One. 2025 Jan 31;20(1):e0317558. doi: 10.1371/journal.pone.0317558 (PMC11785296; doi:10.1371/journal.pone.0317558)
Supplement: S3 Table — (DOCX) [file pone.0317558.s017.docx]

**S3 Table. Univariate analysis of the followed-up patients admitted and not admitted to the ICU after PSM on 4 demographic variables**

| **Variables** | **Total**  **N = 612** | **NO ICU** | **N=306**  **50%** | **ICU** | **N=306**  **50%** |  |
| --- | --- | --- | --- | --- | --- | --- |
|  | **(Mean/N)** | **(Mean/N)** | **(Std/%)** | **(Mean/N)** | **(Std/%)** | **P-value** |
| **Demographics** | **PSM on 4 demographic variables** | | | | | |
| Sex (N, %) |  |  |  |  |  |  |
| Male | 391 | 198 | 64.71% | 193 | 63.07% | 0.674 |
| Female | 221 | 108 | 35.29% | 113 | 36.93% | 0.674 |
| Race (N, %) |  |  |  |  |  |  |
| White | 419 | 210 | 68.63% | 209 | 68.30% | 0.931 |
| Non-White | 193 | 96 | 31.37% | 97 | 31.70% | 0.931 |
| Unknown | 133 | 68 | 22.22% | 65 | 21.24% | 0.769 |
| Ethnicity (N, %) |  |  |  |  |  |  |
| Non-Hispanic | 450 | 227 | 74.18% | 223 | 72.88% | 0.714 |
| Hispanic | 73 | 33 | 10.78% | 40 | 13.07% | 0.383 |
| Unknown | 89 | 46 | 15.03% | 43 | 14.05% | 0.731 |
| Age (Mean, SD) | 62.36 | 62.65 | 16.82 | 62.08 | 16.99 | 0.673 |
| **Co-morbid conditions (N, %)** | | | | | | |
| DM | 195 | 93 | 30.39% | 102 | 33.33% | 0.435 |
| HF | 143 | 64 | 20.92% | 79 | 25.82% | 0.153 |
| CKD | 134 | 69 | 22.55% | 65 | 21.24% | 0.696 |
| COPD | 79 | 37 | 12.09% | 42 | 13.73% | 0.547 |
| HTN | 322 | 152 | 49.67% | 170 | 55.56% | 0.145 |
| CAD | 198 | 97 | 31.70% | 101 | 33.01% | 0.730 |
| Cancer | 120 | 57 | 18.63% | 63 | 20.59% | 0.541 |
| Asthma | 44 | 24 | 7.84% | 20 | 6.54% | 0.532 |
| Psychiatric diagnosis | 338 | 158 | 51.63% | 180 | 58.82% | 0.074 |
| BMI (Mean, SD) | 28.38 | 27.86 | 7.56 | 28.91 | 7.52 | 0.087 |
| **Severity of illness** | | | | | | |
| LOHS (Mean, SD) | 11.53 | 6.28 | 5.89 | 16.78 | 19.46 | **<0.001** |
| ARDS (N, %) | 18 | 0 | 0.00% | 18 | 5.88% | 0.977 |
| Vasopressor (N, %) | 231 | 41 | 13.40% | 190 | 62.09% | **<0.001** |
| Sepsis (N, %) | 113 | 38 | 12.42% | 75 | 24.51% | **<0.001** |
| **AKI_23** | 108 | 23 | 7.52% | 85 | 27.78% | **<0.001** |
| **COVID-19** | 98 | 47 | 15.36% | 51 | 16.67% | 0.660 |
| **Kidney function measures** | | | | | | |
| Baseline eGFR | 89.97 | 87.12 | 28.81 | 92.82 | 29.17 | **0.016** |
| Final eGFR | 80.42 | 80.83 | 29.10 | 80.01 | 27.70 | 0.722 |
| Change in eGFR | -9.55 | -6.29 | 17.11 | -12.81 | 18.81 | **<0.001** |
| Follow-up days | 209.97 | 217.69 | 111.79 | 202.25 | 108.58 | 0.087 |
| eGFR change per year | -19.47 | -12.08 | 37.17 | -26.87 | 42.45 | **<0.001** |
| Fast eGFR decline (N, %) | 388 | 165 | 53.92% | 223 | 72.88% | **<0.001** |

**Legend:** Categorical variables presented as a count with associated percentage, continuous variables presented as value with standard deviation (Std). Univariate logistic p-values < 0.05 were considered significant and have been bolded.

Abbreviations: DM = diabetes mellitus, HF = heart failure, CKD = chronic kidney disease, COPD = chronic obstructive pulmonary disease, HTN = hypertension, CAD = coronary artery disease, BMI = Body Mass Index, LOHS = length of hospital stay, ICU admission = intensive care unit admission, MV = mechanical ventilation, ARDS = acute respiratory distress syndrome, AKI = acute kidney injury, COVID-19 = Corona virus disease 2019, eGFR = estimated glomerular filtration rate.
