# Supplementary material for: Classification and Regression Trees analysis identifies patients at high risk for kidney function decline following hospitalization
Source: PLoS One. 2025 Jan 31;20(1):e0317558. doi: 10.1371/journal.pone.0317558 (PMC11785296; doi:10.1371/journal.pone.0317558)
Supplement: S4 Table — (DOCX) [file pone.0317558.s018.docx]

**S4 Table.** **Logistic regression for fast eGFR decline in the COVID negative subgroup of the PSM matched ICU subset (N = 510).**

| **Variable** |  | **OR (univariable)** | **OR (multivariable)** |
| --- | --- | --- | --- |
| Vasopressor | 1 | 1.81 (1.24-2.66, **) | 1.45 (0.91-2.30) |
| LOHS | Mean (SD) | 1.03 (1.01-1.05, **) | 1.02 (1.00-1.04) |
| ICU admission | 1 | 1.89 (1.31-2.74, ***) | 1.47 (0.96-2.28) |
| AKI_23 | 1 | 1.63 (0.98-2.78) | 1.05 (0.58-1.94) |
| White | 1 | 1.19 (0.80-1.77) | 1.24 (0.81-1.88) |
| Age | Mean (SD) | 0.98 (0.97-0.99, **) | **0.98 (0.97-1.00, *)** |
| Cancer | 1 | 0.96 (0.62-1.49) | 0.97 (0.61-1.57) |
| CKD | 1 | 0.64 (0.42-0.98, *) | 0.69 (0.42-1.13) |
| ARDS | 1 | 0.28 (0.01-2.92) | 0.14 (0.01-1.57) |
| HTN | 1 | 0.72 (0.50-1.03) | 0.69 (0.46-1.03) |
| BMI | Mean (SD) | 1.01 (0.98-1.03) | 1.00 (0.98-1.03) |
| Male | 1 | 0.69 (0.46-1.00) | **0.65 (0.43-0.98, ***) |
| Psychiatric diagnosis | 1 | 1.26 (0.87-1.81) | 1.07 (0.72-1.57) |
| CAD | 1 | 0.90 (0.62-1.33) | 1.25 (0.79-1.98) |

**Legend:**

Abbreviations: LOHS = length of hospital stay, COPD = chronic obstructive pulmonary disease, MV = mechanical ventilation, CKD = chronic kidney disease, HTN = hypertension, DM = diabetes mellitus, CAD = coronary artery disease, eGFR = estimated glomerular filtration rate.

The top variables form Random Forest analysis were selected for Logistic Regression analysis.

P-values < 0.05 were considered significant and were summarized with ‘*’, p-values < 0.01 were considered significant and were summarized with ‘**’, and p-values < 0.001 were considered significant and were summarized with ‘***’.
