# Supplementary material for: Classification and Regression Trees analysis identifies patients at high risk for kidney function decline following hospitalization
Source: PLoS One. 2025 Jan 31;20(1):e0317558. doi: 10.1371/journal.pone.0317558 (PMC11785296; doi:10.1371/journal.pone.0317558)
Supplement: S5 Table — (DOCX) [file pone.0317558.s019.docx]

**S5 Table.** **Logistic regression for fast eGFR decline in the COVID positive subgroup of the PSM matched ICU subset (N = 102).**

| **Variable** |  | **OR (univariable)** | **OR (multivariable)** |
| --- | --- | --- | --- |
| MV days | Mean (SD) | 1.15 (1.04-1.35, *) | 1.03 (0.88-1.35) |
| Age | Mean (SD) | 0.99 (0.96-1.02) | 0.98 (0.94-1.02) |
| MV | 1 | 4.67 (1.61-17.04, **) | 2.03 (0.16-21.00) |
| BMI | Mean (SD) | 0.99 (0.94-1.04) | 0.96 (0.90-1.02) |
| COPD | 1 | 3.44 (0.58-65.87) | 6.10 (0.58-172.30) |
| LOHS | Mean (SD) | 1.05 (1.02-1.10, *) | 1.07 (1.00-1.16) |
| DM | 1 | 2.56 (0.98-7.61) | **8.10 (2.11-37.95, **)** |
| Vasopressor | 1 | 2.38 (0.94-6.64) | 0.25 (0.04-1.30) |
| CKD | 1 | 1.08 (0.39-3.35) | 0.55 (0.13-2.23) |
| Psychiatric diagnosis | 1 | 0.71 (0.30-1.66) | 0.32 (0.09-1.02) |
| ICU admission | 1 | 3.11 (1.31-7.80, *) | 1.84 (0.44-8.63) |
| Hispanic | 1 | 0.71 (0.28-1.82) | 0.34 (0.09-1.14) |
| White | 1 | 0.69 (0.30-1.60) | 0.70 (0.22-2.27) |

**Legend:**

Abbreviations: LOHS = length of hospital stay, COPD = chronic obstructive pulmonary disease, MV = mechanical ventilation, CKD = chronic kidney disease, HTN = hypertension, DM = diabetes mellitus, CAD = coronary artery disease, eGFR = estimated glomerular filtration rate

The top variables form Random Forest analysis were selected for Logistic Regression analysis.

P-values < 0.05 were considered significant and were summarized with ‘*’, p-values < 0.01 were considered significant and were summarized with ‘**’, and p-values < 0.001 were considered significant and were summarized with ‘***’
