# Supplementary material for: Classification and Regression Trees analysis identifies patients at high risk for kidney function decline following hospitalization
Source: PLoS One. 2025 Jan 31;20(1):e0317558. doi: 10.1371/journal.pone.0317558 (PMC11785296; doi:10.1371/journal.pone.0317558)
Supplement: S6 Table — (DOCX) [file pone.0317558.s020.docx]

**S6 Table.** **Univariate analysis of the followed-up patients with and without COVID-19 (N=1,747)**

| **Variables** | **Total** | **N = 1747** | **COVID-19 negative** | | **N = 260 14.88%** | **COVID-19 positive** | **N = 1487 85.12%** |  |
| --- | --- | --- | --- | --- | --- | --- | --- | --- |
| **N = 1747** | **(Mean/N)** | **(Std/%)** | | **(Mean/N)** | **(Std/%)** | **(Mean/N)** | **(Std/%)** | **P-value** |
| **Demographics** | | | | | | | | |
| Sex (N, %) |  |  | |  |  |  |  |  |
| Male | 962 | 55.07% | | 824 | 55.41% | 138 | 53.08% | 0.528 |
| Female | 785 | 44.93% | | 962 | 44.59% | 663 | 46.92% | 0.528 |
| Race (N, %) |  |  | |  |  |  |  |  |
| White | 1270 | 72.70% | | 1105 | 74.31% | 165 | 63.46% | **<0.001** |
| Non-White | 477 | 27.30% | | 382 | 25.69% | 95 | 36.54% | **<0.001** |
| Unknown | 323 | 18.49% | | 258 | 17.35% | 65 | 25.00% | **0.004** |
| Ethnicity (N, %) |  |  | |  |  |  |  |  |
| Non-Hispanic | 1298 | 74.30% | | 1141 | 76.73% | 157 | 60.38% | **<0.001** |
| Hispanic | 189 | 10.82% | | 132 | 8.88% | 57 | 21.92% | **<0.001** |
| Unknown | 260 | 14.88% | | 214 | 14.39% | 46 | 17.69% | 0.199 |
| Age (Mean, SD) | 63.81 | 17.95 | | 63.73 | 18.01 | 64.25 | 17.68 | 0.669 |
| **Co-morbid conditions (N, %)** | | | | | | | | |
| DM | 548 | 31.37% | | 464 | 31.20% | 84 | 32.31% | 0.778 |
| HF | 404 | 23.13% | | 345 | 23.20% | 59 | 22.69% | 0.921 |
| CKD | 396 | 22.67% | | 339 | 22.80% | 57 | 21.92% | 0.818 |
| COPD | 221 | 12.65% | | 193 | 12.98% | 28 | 10.77% | 0.375 |
| HTN | 894 | 51.17% | | 769 | 51.71% | 125 | 48.08% | 0.310 |
| CAD | 543 | 31.08% | | 481 | 32.34% | 62 | 23.85% | **0.008** |
| Cancer | 362 | 20.72% | | 330 | 22.19% | 32 | 12.31% | **<0.001** |
| Asthma | 139 | 7.96% | | 120 | 8.07% | 19 | 7.31% | 0.768 |
| Psychiatric diagnosis | 968 | 55.41% | | 830 | 55.82% | 138 | 53.08% | 0.452 |
| BMI (Mean, SD) | 28.62 | 8.47 | | 28.53 | 8.56 | 29.09 | 7.93 | 0.332 |
| **Severity of illness** | | | | | | | | |
| LOHS (Mean, SD) | 8.44 | 11.22 | | 7.46 | 9.80 | 14.03 | 16.13 | **<0.001** |
| ICU admission (N, %) | 306 | 17.52% | | 255 | 17.15% | 51 | 19.62% | 0.3805 |
| MV (N, %) | 89 | 5.09% | | 57 | 3.83% | 32 | 12.31% | **<0.001** |
| MV days (Mean, SD) | 0.61 | 4.36 | | 0.33 | 2.90 | 2.20 | 8.79 | **<0.001** |
| ARDS (N, %) | 20 | 1.14% | | 3 | 0.20% | 17 | 6.54% | **<0.001** |
| Vasopressor (N, %) | 400 | 22.90% | | 359 | 24.14% | 41 | 15.77% | **0.004** |
| Sepsis (N, %) | 244 | 13.97% | | 175 | 11.77% | 69 | 26.54% | **<0.001** |
| **AKI_23** | 184 | 10.53% | | 139 | 9.35% | 45 | 17.31% | **<0.001** |
| **Fast eGFR decline** | 1076 | 61.59% | | 908 | 61.06% | 168 | 64.62% | 0.309 |
| **Kidney function measures** | | | | | | | | |
| Baseline eGFR | 86.06 | 29.90 | | 85.50 | 29.80 | 89.24 | 30.35 | 0.063 |
| Baseline eGFR > 120 | 189 | 10.82% | | 157 | 10.56% | 32 | 12.31% | 0.466 |
| Baseline eGFR 90 to 120 | 693 | 39.57% | | 580 | 39.00% | 113 | 43.46% | 0.198 |
| Baseline eGFR 60 to 90 | 494 | 28.28% | | 429 | 28.85% | 65 | 25.00% | 0.231 |
| Baseline eGFR 30 to 60 | 292 | 16.71% | | 254 | 17.08% | 38 | 14.62% | 0.372 |
| Baseline eGFR 15 to 30 | 62 | 3.55% | | 52 | 3.50% | 10 | 3.85% | 0.921 |
| Baseline eGFR < 15 | 17 | 0.97% | | 15 | 1.01% | 2 | 0.77% | 0.984 |
| Final eGFR | 77.38 | 30.11 | | 77.50 | 30.10 | 76.68 | 30.21 | 0.686 |
| Change in eGFR | -8.68 | 17.10 | | -8.00 | 16.82 | -12.56 | 18.14 | **<0.001** |
| Follow-up days | 214.33 | 109.28 | | 203.99 | 101.64 | 273.50 | 130.78 | **<0.001** |
| eGFR change per year | -17.03 | 37.68 | | -16.08 | 37.74 | -22.47 | 36.92 | **0.012** |
| **Other lab measures** |  |  | |  |  |  |  |  |
| WBC | 8.03 | 4.19 | | 8.10 | 3.76 | 7.64 | 6.10 | 0.089 |
| Hb | 11.37 | 2.07 | | 11.40 | 2.10 | 11.19 | 1.89 | 0.125 |
| Platelets | 251.65 | 122.42 | | 246.38 | 120.50 | 281.73 | 129.05 | **<0.001** |

**Legend:** Categorical variables presented as a count with associated percentage, continuous variables presented as value with standard deviation (Std). Univariate logistic p-values < 0.05 were considered significant and have been bolded.

Abbreviations: DM = diabetes mellitus, HF = heart failure, CKD = chronic kidney disease, COPD = chronic obstructive pulmonary disease, HTN = hypertension, CAD = coronary artery disease, BMI = Body Mass Index, LOHS = length of hospital stay, ICU admission = intensive care unit admission, MV = mechanical ventilation, ARDS = acute respiratory distress syndrome, AKI = acute kidney injury, COVID-19 = Corona virus disease 2019, eGFR = estimated glomerular filtration rate (mL/min/1.73m^2^), WBC = White Blood Cell count, Hb = Hemoglobin, Platelets = Platelet count.
