# Supplementary material for: Classification and Regression Trees analysis identifies patients at high risk for kidney function decline following hospitalization
Source: PLoS One. 2025 Jan 31;20(1):e0317558. doi: 10.1371/journal.pone.0317558 (PMC11785296; doi:10.1371/journal.pone.0317558)
Supplement: S7 Table — (DOCX) [file pone.0317558.s021.docx]

**S7 Table.** **Univariate analysis of the followed-up patients with and without COVID-19 after PSM on 4 demographic variables**

| **Variables** | **Total**  **N = 520** | **COVID-19 negative** | **N=260**  **50%** | **COVID-19 positive** | **N=260**  **50%** |  |
| --- | --- | --- | --- | --- | --- | --- |
|  | **(Mean/N)** | **(Mean/N)** | **(Std/%)** | **(Mean/N)** | **(Std/%)** | **P-value** |
| **Demographics** | **PSM on 4 demographic variables** | | | | | |
| Sex (N, %) |  |  |  |  |  |  |
| Male | 274 | 136 | 52.31% | 138 | 53.08% | 0.861 |
| Female | 246 | 124 | 47.69% | 122 | 46.92% | 0.861 |
| Race (N, %) |  |  |  |  |  |  |
| White | 341 | 176 | 67.69% | 165 | 63.46% | 0.310 |
| Non-White | 179 | 84 | 32.31% | 95 | 36.54% | 0.310 |
| unknown | 129 | 64 | 24.62% | 65 | 25.00% | 0.919 |
| Ethnicity (N, %) |  |  |  |  |  |  |
| Non-Hispanic | 329 | 172 | 66.15% | 157 | 60.38% | 0.172 |
| Hispanic | 114 | 57 | 21.92% | 57 | 21.92% | 1.000 |
| Unknown | 77 | 31 | 11.92% | 46 | 17.69% | 0.066 |
| Age (Mean, SD) | 65.01 | 65.78 | 17.25 | 64.25 | 17.68 | 0.318 |
| **Co-morbid conditions (N, %)** | | | | | | |
| DM | 169 | 85 | 32.69% | 84 | 32.31% | 0.925 |
| HF | 119 | 60 | 23.08% | 59 | 22.69% | 0.917 |
| CKD | 105 | 48 | 18.46% | 57 | 21.92% | 0.326 |
| COPD | 64 | 36 | 13.85% | 28 | 10.77% | 0.287 |
| HTN | 274 | 149 | 57.31% | 125 | 48.08% | **0.035** |
| CAD | 153 | 91 | 35.00% | 62 | 23.85% | **0.006** |
| Cancer | 97 | 65 | 25.00% | 32 | 12.31% | **<0.001** |
| Asthma | 39 | 20 | 7.69% | 19 | 7.31% | 0.868 |
| Psychiatric diagnosis | 291 | 153 | 58.85% | 138 | 53.08% | 0.185 |
| BMI (Mean, SD) | 28.47 | 27.86 | 7.34 | 29.09 | 7.93 | 0.070 |
| **Severity of illness** | | | | | | |
| LOHS (Mean, SD) | 10.38 | 6.73 | 6.66 | 14.03 | 16.13 | **<0.001** |
| ICU admission (N, %) | 98 | 47 | 18.08% | 51 | 19.62% | 0.654 |
| MV (N, %) | 43 | 11 | 4.23% | 32 | 12.31% | **0.001** |
| MV days (Mean, SD) | 1.20 | 0.19 | 1.02 | 2.20 | 8.79 | **0.003** |
| ARDS (N, %) | 18 | 1 | 0.38% | 17 | 6.54% | **0.005** |
| Vasopressor (N, %) | 111 | 70 | 26.92% | 41 | 15.77% | **0.002** |
| Sepsis (N, %) | 100 | 31 | 11.92% | 69 | 26.54% | **<0.001** |
| **AKI_23** | 68 | 23 | 8.85% | 45 | 17.31% | **0.005** |
| **COVID-19** | 260 | 0 | 0.00% | 260 | 100.00% | 0.999 |
| **Kidney function measures** | | | | | | |
| Baseline eGFR | 87.77 | 86.30 | 27.95 | 89.24 | 30.35 | 0.250 |
| Final eGFR | 77.38 | 78.08 | 28.83 | 76.68 | 30.21 | 0.589 |
| Change in eGFR | -10.39 | -8.22 | 16.25 | -12.56 | 18.14 | **0.005** |
| Follow up days | 238.81 | 204.12 | 84.60 | 273.50 | 130.78 | **<0.001** |
| eGFR change per year | -19.94 | -17.42 | 37.36 | -22.47 | 36.92 | 0.124 |
| Fast eGFR decline (N, %) | 326 | 158 | 60.77% | 168 | 64.62% | 0.365 |

**Legend:** Categorical variables presented as a count with associated percentage, continuous variables presented as value with standard deviation (Std). Univariate logistic p-values < 0.05 were considered significant and have been bolded.

Abbreviations: DM = diabetes mellitus, HF = heart failure, CKD = chronic kidney disease, COPD = chronic obstructive pulmonary disease, HTN = hypertension, CAD = coronary artery disease, BMI = Body Mass Index, LOHS = length of hospital stay, ICU admission = intensive care unit admission, MV = mechanical ventilation, ARDS = acute respiratory distress syndrome, AKI = acute kidney injury, COVID-19 = Corona virus disease 2019, eGFR = estimated glomerular filtration rate.
