# Supplementary material for: Classification and Regression Trees analysis identifies patients at high risk for kidney function decline following hospitalization
Source: PLoS One. 2025 Jan 31;20(1):e0317558. doi: 10.1371/journal.pone.0317558 (PMC11785296; doi:10.1371/journal.pone.0317558)
Supplement: S8 Table — (DOCX) [file pone.0317558.s022.docx]

**S8 Table.** **Logistic regression for fast eGFR decline in the COVID-negative subgroup of the PSM matched COVID-19 subset (N = 260)**

| **Variable** |  | **OR (univariable)** | **OR (multivariable)** |
| --- | --- | --- | --- |
| White | 1 | 1.45 (0.85-2.45) | 1.62 (0.89-2.98) |
| Hispanic | 1 | 0.78 (0.43-1.43) | 0.85 (0.41-1.76) |
| Vasopressor | 1 | 1.90 (1.06-3.49, *) | 1.76 (0.91-3.47) |
| HF | 1 | 0.88 (0.49-1.59) | 1.49 (0.70-3.26) |
| LOHS | Mean (SD) | 1.03 (0.99-1.08) | 1.02 (0.97-1.07) |
| CAD | 1 | 0.85 (0.51-1.43) | 0.94 (0.50-1.76) |
| Psychiatric diagnosis | 1 | 1.14 (0.69-1.90) | 1.15 (0.65-2.01) |
| HTN | 1 | 1.43 (0.87-2.37) | 1.64 (0.91-2.99) |
| Male | 1 | 0.90 (0.54-1.48) | 0.83 (0.48-1.41) |
| COPD | 1 | 0.60 (0.29-1.22) | 0.58 (0.27-1.25) |
| BMI | Mean (SD) | 0.98 (0.95-1.01) | 0.97 (0.93-1.01) |
| Age | Mean (SD) | 1.00 (0.98-1.01) | 0.99 (0.97-1.00) |
| DM | 1 | 0.95 (0.56-1.63) | 1.15 (0.64-2.07) |

**Legend:**

Abbreviations: LOHS = length of hospital stay, COPD = chronic obstructive pulmonary disease, MV = mechanical ventilation, CKD = chronic kidney disease, HTN = hypertension, DM = diabetes mellitus, CAD = coronary artery disease, eGFR = estimated glomerular filtration rate.

The top variables form Random Forest analysis were selected for Logistic Regression analysis.

P-values < 0.05 were considered significant and were summarized with ‘*’, p-values < 0.01 were considered significant and were summarized with ‘**’, and p-values < 0.001 were considered significant and were summarized with ‘***’.
