# Supplementary material for: Classification and Regression Trees analysis identifies patients at high risk for kidney function decline following hospitalization
Source: PLoS One. 2025 Jan 31;20(1):e0317558. doi: 10.1371/journal.pone.0317558 (PMC11785296; doi:10.1371/journal.pone.0317558)
Supplement: S9 Table — (DOCX) [file pone.0317558.s023.docx]

**S9 Table.** **Comparisons between CART decision tree and logistic regression models**

| **Dataset/method** | **Accuracy** | **Sensitivity** | **Specificity** | **AUC** |
| --- | --- | --- | --- | --- |
| Whole cohort, CART | 59.90% | 35.97% | 91.02% | 0.541 |
| Whole cohort, LR | 59.60% | 32.49% | 93.81% | 0.558 |
| COVID-19 negative, CART | 61.15% | 30.89% | 97.99% | 0.559 |
| COVID-19 negative, LR | 59.80% | 36.96% | 91.37% | 0.572 |
| COVID-19 positive, CART | 66.67% | 46.67% | 91.94% | 0.520 |
| COVID-19 positive, LR | 68.63% | 57.78% | 89.91% | 0.595 |

Abbreviations: CART = Classification and Regression Trees, LR = logistic regression, CKD = chronic kidney disease, COVID-19 = Corona virus disease 2019, AUC = Area Under the Receiver Operating Curve.
