# Supplementary material for: Classification and Regression Trees analysis identifies patients at high risk for kidney function decline following hospitalization
Source: PLoS One. 2025 Jan 31;20(1):e0317558. doi: 10.1371/journal.pone.0317558 (PMC11785296; doi:10.1371/journal.pone.0317558)
Supplement: S1 Methods — (DOCX) [file pone.0317558.s024.docx]

**SUPPLEMENTARY METHODS:**

**Data collection and definition of variables:**

All data was collected using the SBUH electronic health record (EHR). Data was automatically fed from the EHR into the “Data Commons COVID-19 registry” at SBUH using a real-time computational method to extract data for analysis. Data on race, ethnicity, age, and gender was obtained from EHR records. Comorbid conditions such as end-stage kidney disease (ESKD), diabetes mellitus (DM), heart failure (HF), chronic kidney disease (CKD), chronic obstructive pulmonary disease (COPD), hypertension (HTN), coronary artery disease (CAD), cancer, and asthma were Identified by the corresponding Internal Classification of Diseases (ICD)-10 codes. “Psychiatric diagnosis” was a term used if patients had ICD codes for any of the following major psychiatric conditions: delirium, medical etiology of psychological symptoms, dementia, neurocognitive disorder, anxiety disorder, stress related disorders (post-traumatic, acute and others), bipolar disorder, depressive disorder, other mood disorder, psychosis, neurodevelopmental disorder, attention deficit hyperactivity disorder or sleep disorders (including sleep apnea, insomnia and others).

Documented height and weight measures at the time of admission were used to calculate body mass index (BMI). The markers of “severity of illness” during hospitalization were determined through ICD-10 codes or documentation in the EHR. These included: length of hospital stay (LOHS), intensive care unit (ICU) admission, mechanical ventilation (MV), the number of MV days, acute respiratory distress syndrome (ARDS), vasopressor use, and sepsis diagnosis. COVID-19 was diagnosed by at least one positive result for severe acute respiratory syndrome coronavirus 2 (SARS-CoV-2) on PCR testing of nasopharyngeal samples. Lastly, patient death was determined through documentation in the EHR.

Most variables in our study for the final cohort are complete, with no missing values. For the three incomplete lab measures, the missing counts (and rates) are as follows: WBC: 2 (0.1%), Hb: 2 (0.1%), and Platelets: 2 (0.1%). Other lab measures had significantly higher percentages of missingness. For these 3 incomplete lab measures, we used mice (Multivariate Imputation by Chained Equations) which is an R package designed to handle missing data through multiple imputations. Given the moderate sample size of 1747 and a low missing rate (<5%), mice package is well-suited because it efficiently handles missing data with statistically sound methods like Predictive Mean Matching (PMM). The main objective of our study was to use clinical measures that are routinely and readable available in the HER. We wanted our model to be simple so it can be easy to validate in other healthcare systems and can be potentially used in clinical applications.

**Acute Kidney Injury (AKI) definition:**

AKI was defined as a rise in serum creatinine (SCr) of ≥1.5 times the baseline to maximum in-hospital SCr based on the KDIGO definition (1). More than 80% of patients did not have a prehospitalization SCr available. Therefore, we used the lowest SCr in the hospital as the “baseline” as has been previously used in COVID-19 studies (2, 3). This approach would not only capture de novo AKI cases in the hospital, but also AKI at the time of hospitalization that subsequently recovered. However, our approach has limitations since the ‘no AKI’ group contained some cases without pre-hospitalization SCr that had AKI at admission but did not have renal recovery. Renal replacement therapy (RRT) was defined as the need for either hemodialysis or continuous kidney replacement therapy, or both.

AKI classes for in-hospital AKI were assigned for each patient based on the KDIGO criteria (1). Class 1 was assigned for an acute increase in SCr by 50% from baseline, Class 2 for an increase by 100%, and Class 3 for an increase of 200% or if the patient required RRT during hospitalization. In this study, we focused on AKI-2/3. A greater than two-fold rise in SCr from baseline is more likely to capture ‘true’ cases of AKI that are more reflective of renal injury. Moreover, moderate/severe AKI is associated with significantly increased health care utilization and costs and poorer long-term patient outcomes including CKD and death (4). Patients requiring acute dialysis during hospitalization were directly assigned to the AKI-3 group and SCr was not used. The lowest and highest SCr were only used for patients who did not require dialysis for AKI during the hospitalization.

**Random Forest (RF) Analysis:**

Random Forest (RF) (5) was used to select the most important variables in classifying the data into ‘with fast eGFR decline’, and ‘without fast eGFR decline’ groups. The *Mean Decrease in Accuracy* (MDA) plots (6) show how much accuracy a RF model loses by excluding each single variable. The decrease in accuracy value is calculated based on an Out of Bag (OOB) (7) dataset by randomly shuffling the data for each variable in the OOB dataset and then subtracting the classification accuracies before and after shuffling. The greater the accuracy loss, the more influential a variable is in generating a good binary classification model. All the variables are presented in descending order by their importance. The *Mean Decrease Gini* (MDG) plots (8) show how much the Gini coefficient loses by excluding each single variable. In classification, the MDG coefficient reflects the contribution of each variable to the homogeneity of nodes and leaves in the generated random forest. The decrease in the Gini coefficient is calculated based on the MDG for each split of the decision node on a specific variable.

In this study, we chose the union of the *top 10 variables* based on the MDA and the MDG coefficient to fit LR and CART decision trees. To avoid overfitting, we trained RF models to do variable selection, keeping only key factors. We applied Breiman’s RF algorithm which is based on Breiman and Cutler’s original Fortran code (9). By default setting, in each RF model, the number of decision trees is 500 and the number of variables used as a potential candidate split variable is 3. Each decision tree in the entire RF is generated based on a random vector sampled independently and with the same distribution. The final RF is a combination of all the trees trained in the forest.

We conducted a sensitivity analysis of the hyperparameters, varying the number of trees (ntree) and the number of variables tried at each split (mtry), and assessed model performance using accuracy and kappa metrics. The results show that accuracy and kappa remain relatively stable across different values of ntree and mtry. However, the combination of ntree = 500 and mtry = 3 achieves a good balance of accuracy (60.56%) and kappa (0.015), which aligns with our initial choice. Increasing ntree beyond 500 does not significantly improve model performance. For example, at ntree = 1000, accuracy and kappa are comparable (accuracy: 59.13%–61.59%, kappa: -0.017–0.038). Similarly, varying mtry slightly affects accuracy and kappa, with mtry = 3 providing an optimal balance between performance and computational efficiency. The sensitivity analysis demonstrates that the model's performance is not highly sensitive to these hyperparameters. Our choice of 500 decision trees and three variables per split (mtry = 3) ensures stable and reasonable performance while maintaining computational efficiency, supporting the robustness of our hyperparameter selection. Additionally, we used the Random Forest model to select influential variables for logistic regression and decision trees. We also compared the variables selected under different combinations of hyperparameters in the Random Forest model, and the results were highly consistent across configurations. This further confirms the reliability of our approach.

Bottom of Form

| **ntree** | **mtry** | **Accuracy** | **Kappa** |
| --- | --- | --- | --- |
| 100 | 2 | 61.19% | -0.003 |
| 100 | 3 | 60.56% | 0.023 |
| 100 | 4 | 58.21% | 0.009 |
| 100 | 5 | 58.62% | 0.038 |
| 200 | 2 | 61.53% | 0.002 |
| 200 | 3 | 60.33% | 0.010 |
| 200 | 4 | 60.10% | 0.051 |
| 200 | 5 | 59.25% | 0.043 |
| 500 | 2 | 61.71% | 0.004 |
| **500** | **3** | **60.56%** | **0.015** |
| 500 | 4 | 58.90% | 0.017 |
| 500 | 5 | 59.01% | 0.037 |
| 1000 | 2 | 61.59% | 0.001 |
| 1000 | 3 | 59.13% | -0.017 |
| 1000 | 4 | 59.48% | 0.028 |
| 1000 | 5 | 59.19% | 0.038 |

We chose the top 10 variables in the Random Forest (RF) analysis rather than using a specific cutoff based on mean decrease in accuracy or Gini. This decision was made because the ranges of these metrics vary significantly across different subsets of the data. For example, the range of mean decrease in Gini for the COVID-matched dataset is between 0 and 40, while for the original dataset, it extends from 0 to 120. Due to these variations, it is impractical to establish a consistent cutoff for all datasets. While we could set different cutoffs for each dataset, we believe that consistently selecting the top 10 variables provides a more straightforward and comparable approach. A smaller number of variables, such as 10 is easier to interpret and present compared to 20 or 30. This makes it more straightforward to communicate the most important predictors to stakeholders or collaborators, especially in clinical or practical settings where simplicity is valued.

**Logistic Regression (LR):**

Logistic Regression (LR) is a parametric model which can help identify the significant effects and the corresponding effect direction from the estimated odds ratios (ORs) and p-values (10). After variable selection through RF, we fitted multivariate LR models to determine the significant risk factors associated with post-hospitalization eGFR decline. The ORs were calculated as the probability of having fast eGFR decline divided by the probability of not having fast eGFR decline and were then subjected to a logit transformation. LR was estimated via maximum likelihood estimation (MLE) (11) with the logit transformed ORs as the dependent or response variable and the selected clinical measures as the independent variables or predictors. From the estimated OR and the corresponding p-value for each independent variable, we determined the influential clinical measures and the direction of their effect on fast eGFR decline.

**Classification and Regression Trees (CART) analysis:**

To help understand that effect of each clinical measure, we used a non-parametric and non-linear supervised learning algorithm called CART “Classification and Regression Trees’. CART were first reported by Leo Breiman, Jerome Friedman, Richard Olshen, and Charles Stone in 1984 (12). This type of decision tree algorithm utilizes Gini purity to identify the ideal attributes on which to split each decision node. A CART decision tree starts with a *root node* which contains all the patients and employs a divide-and-conquer strategy by conducting a greedy search to identify the optimal split points. The outgoing branches from the root node are then split into the internal nodes, also known as *decision nodes,* which contain subsets of patients. Based on the variables we analyze, both node types conduct evaluations from homogenous subsets. As a decision tree grows, it becomes increasingly difficult to keep its purity. This is known as data fragmentation and can lead to overfitting. To avoid overfitting, we first conducted RF using all the variables and then ranked the importance of the variables to select several key features for predicting our target variables (binary fast vs not fast eGFR decline). In our CART decision trees, the number of observations in a terminal node had to be at least 2% of the sample size. The maximum depth of the decision tree was set to be 3.

**Comparison between Logistic Regression and CART decision tree:**

Unlike other machine learning methods, which can only be used to do prediction, LR and CART allow us to understand the effect and relative impact of the independent variables on the dependent variable of interest in study, i.e. fast eGFR decline. In this study, for each subset, all patients were randomly split into two parts, 80% for training and 20% for testing. The training set was used to train the multivariate LR and CART decision tree models, and then the model performance was evaluated on the testing set. The accuracy, sensitivity, specificity and AUC (Area Under the Receiver Operating Curve) are reported in **S9 Table**. The AUC (13) was used as a summary of the model's power to correctly rank a randomly chosen positive instance higher than a randomly chosen negative one. It is not influenced by the decision threshold or the imbalance of the classes in the dataset, making it a valuable metric for comparing models. (**S9 Table**).

**SUPPLEMENTARY REFERNCES:**

1. KDIGO Clinical Practice Guideline for Acute Kidney Injury. Kidney International. 2012;2

(1):1-138.

2. Fisher M, Neugarten J, Bellin E, Yunes M, Stahl L, Johns TS, et al. AKI in Hospitalized Patients with and without COVID-19: A Comparison Study. J Am Soc Nephrol. 2020;31(9):2145-57.

3. Sun S, Annadi RR, Chaudhri I, Munir K, Hajagos J, Saltz J, et al. Short- and Long-Term Recovery after Moderate/Severe AKI in Patients with and without COVID-19. Kidney360. 2022;3(2):242-57.

4. Chao CT, Tsai HB, Wu CY, Lin YF, Hsu NC, Chen JS, et al. The severity of initial acute kidney injury at admission of geriatric patients significantly correlates with subsequent in-hospital complications. Sci Rep. 2015;5:13925.

5. Breiman L. Random forests. Machine learning. 2001;45:5-32.

6. Louppe G, Wehenkel L, Sutera A, Geurts P. Understanding variable importances in Forests of randomized trees2013.

7. Bylander T. Estimating generalization error on two-class datasets using out-of-bag estimates. Machine learning. 2002;48:287-97.

8. Martinez-Taboada F, Redondo JI. The SIESTA (SEAAV Integrated evaluation sedation tool for anaesthesia) project: Initial development of a multifactorial sedation assessment tool for dogs. PLoS One. 2020;15(4):e0230799.

9. Livingston F. Implementation of Breiman’s random forest machine learning algorithm. ECE591Q Machine Learning Journal Paper. 2005:1-13.

10. Bender R, Grouven U. Ordinal logistic regression in medical research. J R Coll Physicians Lond. 1997;31(5):546-51.

11. Breslow NE, Holubkov R. Maximum likelihood estimation of logistic regression parameters under two‐phase, outcome‐dependent sampling. Journal of the Royal Statistical Society: Series B (Statistical Methodology). 1997;59(2):447-61.

12. Breiman L, Friedman J, Stone CJ, Olshen RA. Classification and Regression Trees: Taylor & Francis; 1984.

13. DeLong ER, DeLong DM, Clarke-Pearson DL. Comparing the Areas under Two or More Correlated Receiver Operating Characteristic Curves: A Nonparametric Approach. Biometrics. 1988;44(3):837-45.
