# Supplementary material for: Classification and Regression Trees analysis identifies patients at high risk for kidney function decline following hospitalization
Source: PLoS One. 2025 Jan 31;20(1):e0317558. doi: 10.1371/journal.pone.0317558 (PMC11785296; doi:10.1371/journal.pone.0317558)
Supplement: Attachment — Submitted filename: FK edits Kidney ML paper - Author Responses Letter 121224.docx [file pone.0317558.s025.docx]

Dear Dr. Hosohata and the PLOS ONE editorial board,

Thak you for your feedback and the critiques and comments and for providing us with the opportunity to improve our manuscript. Thank you to the two reviewers for their time and important feedback.

Please see our point-by-point response to the Editor and Reviewer comments/suggestions below:

We sincerely hope that our changes based on these suggestions have significantly improved our manuscript and will be considered acceptable for publication.

Thank you!

Farrukh. M. Koraishy, MD, PhD, FASN

Professor of Nephrology/Medicine, Stony Brook University, School of Medicine

**Editor:**

1. Please ensure that your manuscript meets PLOS ONE's style requirements, including those for file naming. The PLOS ONE style templates can be found at https://journals.plos.org/plosone/s/file?id=wjVg/PLOSOne_formatting_sample_main_body.pdf and <https://journals.plos.org/plosone/s/file?id=ba62/PLOSOne_formatting_sample_title_authors_affiliations.pdf>

**Author response:** Thank you for pointing out this error on our part. We have reviewed both the guidelines and adjusted the formatting of the manuscript accordingly.

2. Please note that PLOS ONE has specific guidelines on code sharing for submissions in which author-generated code underpins the findings in the manuscript. In these cases, we expect all author-generated code to be made available without restrictions upon publication of the work. Please review our guidelines at https://journals.plos.org/plosone/s/materials-and-software-sharing#loc-sharing-code

and ensure that your code is shared in a way that follows best practice and facilitates reproducibility and reuse.

**Author response:** Yes, we will ensure that our code is shared in a way that follows best practice and facilitates reproducibility and reuse. The complete source code for this project is publicly available in the GitHub repository. This allows for full transparency of the research methodology, reproducibility of the analysis, and collaboration and peer review

GitHub: <https://github.com/cutsuper/Trees-analysis-identifies-patients-at-high-risk-for-kidney-function-decline>

3. Please include a caption for figure 6, 7, 8, 9, 10, 11, 12.

**Author response**: We have 5 main and 12 supplementary figures. All of these include a title and legend.

4. We note you have included a table to which you do not refer in the text of your manuscript. Please ensure that you refer to Table 2 in your text; if accepted, production will need this reference to link the reader to the Table.

**Author response:** We have 4 main tables; all of these are included in the text when they are cited. They all have titles and legends.

5. Please review your reference list to ensure that it is complete and correct. If you have cited papers that have been retracted, please include the rationale for doing so in the manuscript text, or remove these references and replace them with relevant current references. Any changes to the reference list should be mentioned in the rebuttal letter that accompanies your revised manuscript. If you need to cite a retracted article, indicate the article’s retracted status in the References list and also include a citation and full reference for the retraction notice.

**Author response:** We double checked and confirmed that none of the papers we cited have been retracted. Thank you.

**Reviewer #1:**

We thank Reviewer 1 for the valuable critiques and suggestions. Below are our point-by-point responses.

Introduction

1. The authors should report the strong relationship between CKD and AKI, where one type of kidney injury can predispose to the other in both directions and both ways.

**Author response:** Thank you for pointing this out. We have added this in the Introduction section along with a relevant reference.

2. The authors should briefly report the frequency of AKI in hospitalized patients with COVID-19 and without COVID-19 to better contextualize.

**Author response:** Thank you again for this important suggestion. We have updated the Introduction section to reflect this information along with relevant references.

Methods

3. The researchers should be able to classify kidney injury by the time of onset, such as AKI lasting up to 7 days, AKD between 7 and 30 days, and CKD lasting more than 30 days.

**Author response:** We agree this is an important classification. Unfortunately, we do not have serial serum creatinine to make this determination in our dataset.

4. The authors analyzed a lot of clinical data to understand the progression of CKD after COVID-19. However, it would be interesting to also analyze clinical laboratory data, such as complete blood count, to understand the relationship between hemoglobin concentration, leukocyte count, and platelet count with the progression to CKD.

**Author response:** Thank you for this important suggestion. We included the last recorded measures during hospitalization of WBC, Hb, and platelet count in the univariate analysis table alongside other lab measures (Please see updated Table 1 and new Supplementary Table 6). Hb level and platelet count were significantly different across the eGFR decline groups, while platelet count was significantly different across the COVID-19 groups. However, when added to the logistic regression analysis, none of them were significant in the multivariable logistic regression model. Therefore, we did not include them in subsequent analyses.

5. Was Machine Learning analysis able to observe other clinical factors related to greater fast GFR decline in severe-ill patients, such as mechanical ventilation requirements, or low urinary output?

**Author response:** Mechanical ventilation (MV) was included in the models. Unfortunately, we did not have accurate urine output data to estimate AKI. This is an important point, and we have added this to the Limitations paragraph.

6. Did the researchers perform a comparative analysis of eGFR at hospital admission between patients with and without COVID-19? Moreover, within the sample of patients with COVID-19. Was there a comparison of eGFR between patients with rapid loss of renal function and patients without rapid loss of renal function?

**Author response:** We appreciate this important advice. We created a new univariate table comparing patients with and without COVID-19 in the overall cohort (please see new Supplementary Table 6). Baseline eGFR did not differ significantly between COVID-19 and non-COVID-19 patients. Longitudinal analysis showed that yearly eGFR changes were significantly different between COVID-19 and non-COVID-19 patients, indicating that COVID-19 may influence kidney function decline over time

| Mean egfr change per year by COVID status | |
| --- | --- |
| Non-COVID | **COVID-19** |
| -16.08 mL/min/1.73m2 per year | **-22.47** mL/min/1.73m^2^ per year |

The new table and these findings are referred to in the updated Results section #3.

Discussion

7. Researchers should engage in a thorough discussion of our findings that 'patients with fast eGFR decline were more likely to have a higher baseline eGFR.' This intriguing contradiction challenges the existing literature, which suggests that CKD (lower GFR) is associated with a greater predisposition to AKI and that the presence of AKI is associated with progression or may even cause CKD.

**Author response:** Thank you for highlighting this interesting finding that certainly warranted more mention. There is a significant difference in baseline eGFR between patients with and without rapid eGFR decline, with higher baseline eGFR observed in those experiencing faster decline. In our updated Table 1 and new Supplementary Table 6, we have sub-divided baseline GFR in multiple categories based on the CKD stages classification ('>120', '90 to 120', '60 to 90', '30 to 60', '15 to 30', and '<15') and performed univariate analysis on each baseline eGFR group. Patients with fast eGFR decline show a significantly higher proportion of baseline eGFR > 120 mL/min/1.73m^2^ and a substantially lower proportion of baseline eGFR < 60. mL/min/1.73m^2^

| **Variables** | **Total** | **N = 1747** | **Without Fast eGFR decline** | **N = 671 38.41%** | **Fast eGFR decline** | **N = 1076 61.59%** |  |
| --- | --- | --- | --- | --- | --- | --- | --- |
| **N = 1747** | **(Mean/N)** | **(Std/%)** | **(Mean/N)** | **(Std/%)** | **(Mean/N)** | **(Std/%)** | **P-value** |
| Baseline eGFR | 86.06 | 29.90 | 78.91 | 29.12 | 90.51 | 29.52 | <0.001 |
| Baseline eGFR > 120 | 189 | 10.82% | 34 | 5.07% | 155 | 14.41% | <0.001 |
| Baseline eGFR 90 to 120 | 693 | 39.57% | 254 | 37.85% | 439 | 40.80% | 0.241 |
| Baseline eGFR 60 to 90 | 494 | 28.28% | 204 | 30.40% | 290 | 26.96% | 0.133 |
| Baseline eGFR 30 to 60 | 292 | 16.71% | 134 | 19.97% | 158 | 14.68% | 0.005 |
| Baseline eGFR 15 to 30 | 62 | 3.55% | 32 | 4.77% | 30 | 2.79% | 0.041 |
| Baseline eGFR < 15 | 17 | 0.97% | 13 | 1.94% | 4 | 0.37% | 0.003 |

We have added this finding to the Results section #1. In the Discussion section, we have tried to provide potential explanations of these findings based on previous studies that have shown that above normal GFR might reflect glomerular hyperfiltration and great risk for CKD progression. While did not find a difference in CKD diagnosis (please see Table 1) in the eGFR decline groups, it’s not clear why patients with low baseline eGFR might have lower relative eGFR decline, although the number of patients with eGFR < 60 mL/min/1.73m^2^ in our study was low (only 21% of the cohort). This interesting association of baseline eGFR with eGFR decline certainly needs further exploration in research studies.

8. Researchers should discuss in more detail the possibility of using CART in clinical practice, especially in ICUs, as patients are more severely ill and have a higher frequency of AKI. And how useful could this tool be for physicians when assessing these patients.

**Author response:** Thank you again for this excellent suggestion. We have added the following para in the Discussion section. We have also added relevant references in the text.

‘The application of CART models in clinical practice, particularly in ICU settings, offers several advantages (60, 61). CART’s ability to generate simple and interpretable decision trees makes it a practical tool for risk stratification and decision-making in high-risk environments. For ICU patients, who often experience a higher frequency of AKI due to severity illness and other nephrotoxic insults and consequently have a high risk of post-discharge GFR decline and CKD progression. CART can help identify high-risk individuals early, allowing for targeted interventions such as nephroprotective measures or closer monitoring. Furthermore, CART’s flexibility in handling complex interactions between variables, such as baseline renal function, comorbidities, laboratory parameters and treatment modalities, is particularly relevant for ICU populations. Integrating CART models into electronic health record systems could facilitate real-time risk assessments, improving patient outcomes (62, 63). However, successful implementation requires rigorous validation of the model in diverse ICU settings and the availability of high-quality, real-time data. This should be the focus of future validation and implementation studies.’

**Reviewer #2:**

We thank Reviewer 2 for the valuable critiques and suggestions. Below are our point-by-point responses.

1. The decision to remove variables with more than 5% missing data might lead to loss of potentially important information. It could be beneficial to explore and justify additional methods to handle missing data, such as multiple imputations.

**Author response:** Thank you for this suggestion. All the previously included variables in our paper for the final cohort are complete, with no missing value. For the three additional lab measures that we added based on Reviewer #1’s advice, the missing counts (rates) are as follows: WBC: 2 (0.1%), Hb: 2 (0.1%), and Platelets: 2 (0.1%). Other lab measures had a significantly higher % of missingness. For these 3 new variables, we used mice (Multivariate Imputation by Chained Equations) which is an R package designed to handle missing data through multiple imputations. Given the moderate sample size of 1747 and a low missing rate (<5%), mice package is well-suited because it efficiently handles missing data with statistically sound methods like Predictive Mean Matching (PMM).

The above part was added to the Supplementary Methods.

The main objective of our study was to use clinical measures that are routinely and readable available in the HER. We wanted our model to be simple so it can be easy to validate in other healthcare systems and can be potentially used in clinical applications.

2. Detail whether the matching was performed one-to-one, one-to-many, or another method, as this impacts the analysis.

Author response: Thank you for this important comment. We have added the following passage in the PSM Methods section to explain this better.

Propensity score matching (PSM) was conducted using **nearest neighbor matching in a 1:1 ratio without replacement**. Propensity scores were estimated through logistic regression based on the important demographic covariates: sex, race, ethnicity and age. This method ensures that each treated unit (ICU admission or COVID-19 positive) is matched to a control unit (non-ICU admission or COVID-19 negative respectively) with the closest propensity score, thereby minimizing group differences and enabling direct comparisons.

3. Describe how balance was assessed post-matching, as this is crucial to ensure that the groups are comparable.

**Author response:** Thank you again for this important point. We have added the following passage in the PSM Methods section to explain this better.

To assess balance, we calculated **absolute standardized mean differences (SMDs)** for both the unadjusted (pre-matching) and adjusted (post-matching) datasets. The absolute mean difference plots demonstrate that all SMDs post-matching decreased to below 0.10, which is a commonly used threshold for acceptable balance, as previously reported (33). This substantial reduction in SMDs indicates a significant improvement in balance after matching, ensuring the comparability of the matched groups (Supp Fig Xa and b).

The following figures have been added to the Supplementary file.

Supp Fig Xa. Standardized Mean Differences (SMDs) Before and After Matching for ICU Admission Groups

Supp Fig Xb. Standardized Mean Differences (SMDs) Before and After Matching for COVID-19 Diagnosis Groups

4. The rationale for selecting 500 decision trees and three variables for RF models could be more detailed. Also, discuss the sensitivity of results to different hyperparameter choices.

**Author response:** Thank you for this valid advice. We have added the following to the Supplementary Methods section to explain this better.

We conducted a sensitivity analysis of the hyperparameters, varying the number of trees (ntree) and the number of variables tried at each split (mtry), and assessed model performance using accuracy and kappa metrics. The results show that accuracy and kappa remain relatively stable across different values of ntree and mtry. However, the combination of ntree = 500 and mtry = 3 achieves a good balance of accuracy (60.56%) and kappa (0.015), which aligns with our initial choice. Increasing ntree beyond 500 does not significantly improve model performance. For example, at ntree = 1000, accuracy and kappa are comparable (accuracy: 59.13%–61.59%, kappa: -0.017–0.038). Similarly, varying mtry slightly affects accuracy and kappa, with mtry = 3 providing an optimal balance between performance and computational efficiency. The sensitivity analysis demonstrates that the model's performance is not highly sensitive to these hyperparameters. Our choice of 500 decision trees and three variables per split (mtry = 3) ensures stable and reasonable performance while maintaining computational efficiency, supporting the robustness of our hyperparameter selection. Additionally, we used the Random Forest model to select influential variables for logistic regression and decision trees. We also compared the variables selected under different combinations of hyperparameters in the Random Forest model, and the results were highly consistent across configurations. This further confirms the reliability of our approach.

Bottom of Form

| **ntree** | **mtry** | **Accuracy** | **Kappa** |
| --- | --- | --- | --- |
| 100 | 2 | 61.19% | -0.003 |
| 100 | 3 | 60.56% | 0.023 |
| 100 | 4 | 58.21% | 0.009 |
| 100 | 5 | 58.62% | 0.038 |
| 200 | 2 | 61.53% | 0.002 |
| 200 | 3 | 60.33% | 0.010 |
| 200 | 4 | 60.10% | 0.051 |
| 200 | 5 | 59.25% | 0.043 |
| 500 | 2 | 61.71% | 0.004 |
| **500** | **3** | **60.56%** | **0.015** |
| 500 | 4 | 58.90% | 0.017 |
| 500 | 5 | 59.01% | 0.037 |
| 1000 | 2 | 61.59% | 0.001 |
| 1000 | 3 | 59.13% | -0.017 |
| 1000 | 4 | 59.48% | 0.028 |
| 1000 | 5 | 59.19% | 0.038 |

5. Ensure the selection of influential factors in multivariate LR and CART analyses is clearly rationalized, possibly by exploring whether other thresholds for variable inclusion could impact findings.

**Author response:** Thank you again for this valid advice. We have added the following to the Supplementary Methods section to explain this better.

We chose the top 10 variables in the Random Forest (RF) analysis rather than using a specific cutoff based on mean decrease in accuracy or Gini. This decision was made because the ranges of these metrics vary significantly across different subsets of the data. For example, the range of mean decrease in Gini for the COVID-matched dataset is between 0 and 40, while for the original dataset, it extends from 0 to 120. Due to these variations, it is impractical to establish a consistent cutoff for all datasets. While we could set different cutoffs for each dataset, we believe that consistently selecting the top 10 variables provides a more straightforward and comparable approach. A smaller number of variables, such as 10 is easier to interpret and present compared to 20 or 30. This makes it more straightforward to communicate the most important predictors to stakeholders or collaborators, especially in clinical or practical settings where simplicity is valued.

6. Ensure consistent use of terms and abbreviations throughout the text. For instance, if "eGFR" is used, avoid switching to "GFR" without explanation.

**Author response:** Thank you for pointing this out. We have reviewed the files again and ensured this is addressed.

7. Use consistent formatting for statistical data, such as percentages and p-values, to maintain uniformity.

**Author response:** Thank you for this advice. We have made the updates in the revised version. We consistently used integers for counting numbers, two decimal places for means, standard deviations, odds ratios, proportions (as percentages), and accuracy percentages. Three decimal places were used for p-values, AUC, and Kappa metrics. For p-values less than 0.0005 that cannot be rounded up to 0.001, we represented them as "<0.001."
